# Young men and young women in secure care: gender differences in the placement of those with mental health needs

**DOI:** 10.1186/s12888-021-03440-7

**Published:** 2021-09-03

**Authors:** Annie Bartlett, Jared G. Smith, Louise Warner, Heidi Hales

**Affiliations:** 1 IMBE, St George’s, University of London, London, UK; 2 Population Health Research Institute, St George’s, University of London, London, UK; 3 CNWL NHS Foundation Trust, London, UK; 4 Adolescent Forensic Psychiatry, West London NHS Trust, London, UK

**Keywords:** Gender, Secure care, Mental health, Youth justice, Welfare, Equality act

## Abstract

**Background:**

The system of secure care for young people in England and Wales comprises youth justice, welfare and mental health facilities. Empirical studies have failed to investigate the system as a whole. The National Adolescent Study in 2016 was the first to provide comprehensive system wide information. This paper, derived from that data set, addresses equity of service provision for young men and women in secure care who have mental health problems.

**Methods:**

The detained census population of English young people in 2016 was 1322 and detailed data were available on 93% of this population, including 983 young men and 290 young women. The descriptive census data were interrogated to identify associations between gender, other sociodemographic and clinical variables, using Chi-square and Fisher’s exact tests.

**Results:**

Numerically more young men in secure care than young women in secure care warrant a psychiatric diagnosis but young women had a 9 fold increase in the odds of having a diagnosis compared with the young men. The pattern of mental health diagnoses differed significantly by gender as did the legislative framework under which females and males were placed. This different pattern of secure care placement continued to differ by gender when the nature of the mental health diagnosis was taken into account.

**Conclusions:**

No definitive explanation is evident for the significantly different placement patterns of young men and young women with the same mental health diagnoses, but the anticipated consequences for some, young men and some young women are important. Proper explanation demands an examination of process variables outwith the remit of this study. The lack of routine scrutiny and transparent processes across secure settings could be responsible for the development of these differential placement practices; these practices seem at odds with the duty placed on public services by the Equality Act.

## Background

**Fig. 1:**
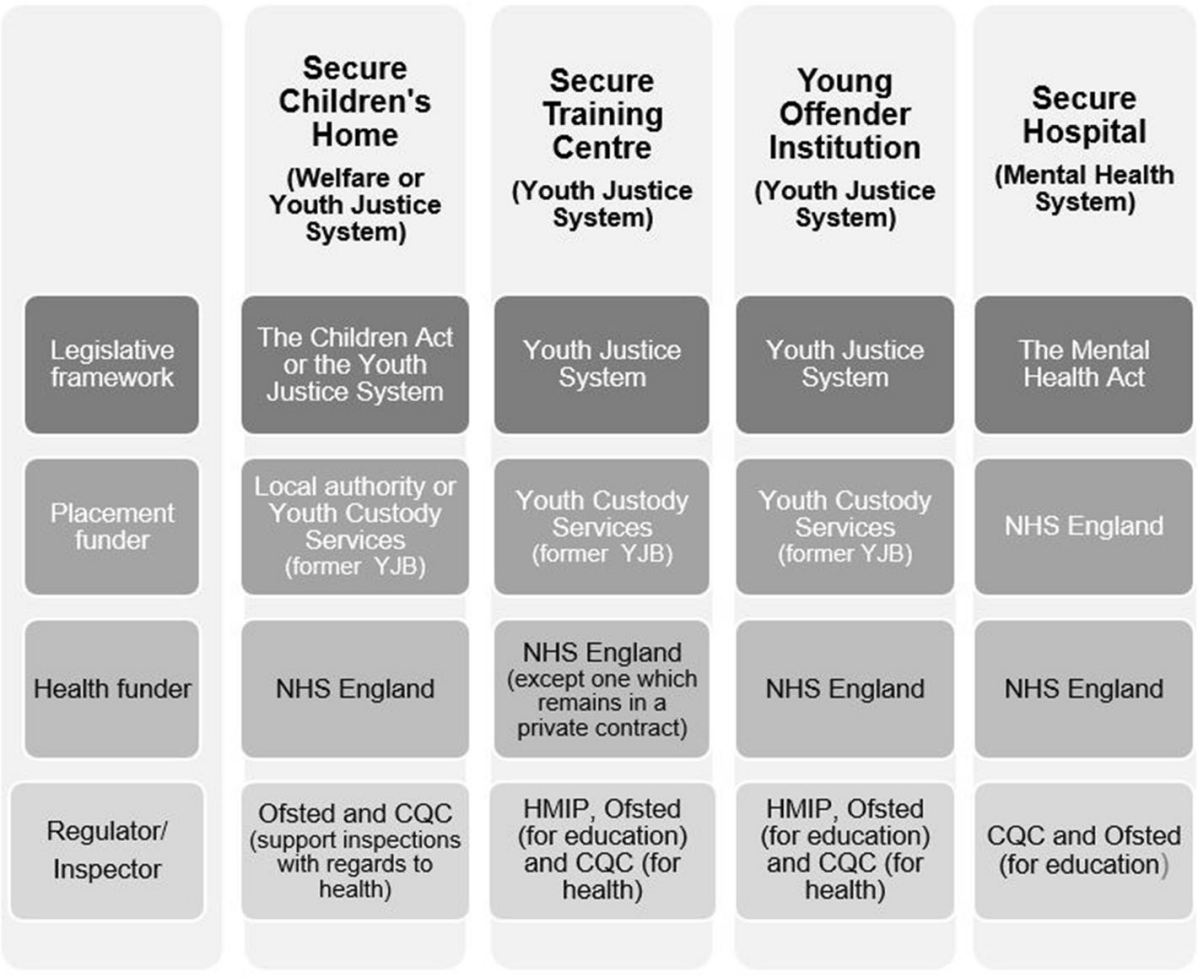
The secure estate for young people in England. Please note: NHS = National Health Service; YCS = Youth Custody Services; CQC = Care Quality Commission; HMIP = Her Majesty’s Chief Inspector of Prisons

The current tripartite, secure system of care for Young People (YP) in England and Wales is complex (see Fig. 1 below). No policy review or empirical project had simultaneously considered all three components of the system until 2016 when the National Adolescent Study [1] found significant and varied rates of mental health problems in young people in different parts of the system. A remaining gap in available information is comparative data on the mental health of young men (YM) and young women (YW) across the tripartite system as a whole.

The system of secure care in England and Wales (see Fig. 1), as elsewhere [2, 3] involves multiple types of unit, differing levels of security and several separate bodies of legislation. It comprises secure hospital units (high dependency units, low and medium secure units), secure children’s homes and youth justice facilities (Young Offender Institutions and Secure Training Centres). It is managed, inspected and run by different agencies. It shares the common purpose of detention of a young person but the explicit ethos of the mental health, welfare and youth justice facilities varies, as the terms imply. This somewhat masks the overlapping tasks of staff working in such units who will all have, to some degree, responsibilities for mental health, child welfare and security. Minimum ages of detention differ in the different kinds of unit and detention is for highly variable time frames, sometimes of unspecified duration. Recent UK reviews [4–6] have noted the involvement of some young people with multiple components of the overall system highlighting the organisational interdependency of the provider units and the need for strong multi-agency arrangements prior to detention [7]. Children from care backgrounds are overrepresented in the youth justice system [8]. Young people detained under the Mental Health Act in secure hospitals will often have gone through the custodial, youth justice system. Young people who enter youth custody on remand become “Looked after Children” under the welfare system, even if they were not previously, until the point of sentence. Those who spend more than 13 weeks in prison acquire “Leaving Care” status on release.

Empirical material is available on UK Young People’s mental health in all three components of the system but these data are only available by separate systemic components i.e. child welfare [9–11], secure hospital care [12–14] and the Youth Justice System (YJS) [15, 16]; this single agency perspective is also evident in other jurisdictions [17]. Relevant UK based studies describe units in which there are more young men than young women and high levels of mental health difficulty, variably defined. Seventy percent of young people in residential care had a mental health problem [11], with young men more likely to do so than young women. One third of young offenders had detectable mental health needs, with young women more likely than young men to do so by a factor of 1.5 [15]. Patterns of morbidity within secure mental health units varied by gender with young men more likely to suffer from psychosis and neurodevelopmental disorders and young women from emerging emotionally unstable personality disorder [14]. Comparisons are frequently made to community samples rather than to other types of institutional care. The specifics of jurisdictions can also limit the value of international data comparisons. Within jurisdictions rapid changes in the nature and size of services can compromise analysis [18].

Single agency review, particularly of youth justice facilities e.g. [19] can also lead to the partial or complete neglect of young women who, even if considered, may be present in only small numbers in individual studies, regardless of the site of research [20, 21]; pooling of data may be required to produce meaningful findings [22].

Therefore, this paper compares mental health problems in young men and young women. The paper relies on data obtained in the National Adolescent Study [1] and uses additional analysis to consider the extent to which this system has been capable of providing services that are fair to young men and to young women with mental health problems. It starts from the premise that regardless of the size of a population, in this instance a population defined by a protected characteristic i.e. gender, that population has the right to the same quality of services, a principle established in the 2010 Equality Act.

To do this, it initially explores the distribution and capacity of secure services in England and Wales by gender and the mental health morbidity of young men and women detained therein. Further analysis then considers the extent to which the placement patterns of young men and young women are appropriately linked to their mental health problems i.e. whether or not they are in the right place to get relevant clinical care.

## Methods

The National Adolescent Study [1, 23] described facilities for detained, English, young people and characterized those young people who were detained on one day in 2016. A comprehensive account of the methods employed to generate census data and the approach taken to the basic analysis are to be found in Appendix B of the National Adolescent Study Census report [1]. The following information is salient to this paper:

All data on individuals were anonymized at the site of detention so that investigators only received anonymous data. Data sheets included information on the young person’s gender, age and ethnicity; geographical origin; legislation under which they were detained; number and type of previous secure and open placements (care facilities, mental hospital and youth justice placements); previous contact with community services (mental health, Youth Offending Teams, Local Authority services); clinical needs (physical and mental); risk to self and others.

Data sheets were filled in by a range of professionals, sometimes but not invariably mental health clinicians. The level of detail varied and diagnostic systems were not invariably in use within all types of unit. All mental health information (including mental health needs, treatment, diagnoses and risks) was considered by HH (Consultant in Adolescent Forensic Psychiatry) against established ICD 10 diagnostic criteria. The level of co-morbidity in the population required assessment of the most important clinical need, which was designated the primary diagnosis. The designation took into account pathways of care, acuity of illness and non-episodic disorders e.g. personality disorder and neurodevelopmental disorders and was ultimately a clinical judgement. The data sheet used in the census and full details on the rules governing that process of diagnostic decision making are available in Appendix B pages 37–44 of the National Adolescent Study Census report [1] Missing data was noted where there was absent information about any mental health needs.

### Statistical analyses

Frequencies and percentages were used to present descriptive data. Associations between gender and sociodemographic and clinical variables (e.g. psychiatric status) were investigated with Chi-square and Fisher’s exact tests. Where appropriate, odds ratios (ORs) and 95% confidence intervals (CIs) were calculated for binary factors. To control for Type 1 errors due to multiple comparisons, the false discovery rate (FDR) approach, controlled at level α = 5% [24], was applied to each set of analyses in determining statistical significance. Statistical analyses were completed with SPSS (IBM, Version 25.0).

## Results

On the census day (14.09.16), 1322 English young people were detained in secure care; 1260 (95.3%) were placed in England and 62 (4.7%) placed in Wales or Scotland. Data was sourced from 3 High Dependency Units (HDUs), 10 Psychiatric Intensive Care Units (PICUs), 9 Low Secure Units, 7 Medium Secure Units, 19 Secure Children’s Homes (SCHs), 3 Secure Training Centres (STCs) and 5 Young Offender Institutions (YOIs). Just over three quarters of available placements in England were in use on the day of the census. Of the detained young people, 983 (76.9%) were young men, 290 (22.7%) were young women, 5 (0.4%) individuals identified as transgender and 1 (0.1%) as intersex. Data cited below are derived from census questionnaires which were received on 93% of the 1322 young people. There were high response rates, > 85%, across all kinds of unit, except the small number of High Dependency hospital units.

Sociodemographic comparisons of the characteristics of young men and young women in this population are given in Table 1. They are significantly different in terms of age, ethnicity and the presence of any physical illness or disability. Specifically (see Table 1 for additional detail), a larger proportion of the young women detained are aged 15 years or less, and many more of the young men come from a Black and Minority Ethnic Group (BAME) background (a four-fold increase in odds).

**Table 1:** Demographic and clinical characteristics of female and male young people in secure care. Values represent frequencies (percentages) unless otherwise stated

	Females	Males	Females vs Males	
			*p*	OR (95% CI)
Age	*n* = 239	*n* = 899		
Mean years (SD, range)	**15.9 (1.3,12–22)**	**16.5 (1.0,12–18)**	**< 0.001**	
	*n*(%)	*n*(%)		
< = 15 years	**82 (34.3)**	**145 (16.1)**		
16–17 years	**146 (61.1)**	**670 (74.5)**		
18 + years	**11 (4.6)**	**84 (9.3)**	**< 0.001**	
Ethnicity	*n* = 240	*n* = 838		
White	**202 (84.2)**	**481 (57.4)**		
Black	**9 (3.8)**	**183 (21.8)**		
Asian	**8 (3.3)**	**51 (6.1)**		
Mixed/Other	**21 (8.8)**	**123 (14.7)**	**< 0.001**	
BAME	**38 (15.8)**	**357 (42.6)**	**< 0.001**	**0.25 (0.18,0.37)**
Physical disability/illness	*n* = 218	*n* = 838		
One or more	**41 (18.8)**	**219 (26.1)**	**0.025**	**0.66 (0.45,0.95)**
Psychiatric Diagnosis	*n* = 244	*n* = 854		
None	**30 (12.3)**	**482 (56.4)**		
One	**95 (38.9)**	**206 (24.1)**		
Two	**77 (31.6)**	**94 (11.0)**		
Three	**31 (12.7)**	**51 (6.0)**		
Four to five	**11 (4.5)**	**21 (2.5)**	**< 0.001**	
Any diagnosis	**214 (87.7)**	**372 (43.6)**	**< 0.001**	**9.24 (6.16,13.86)**
Three or more	**42 (17.2)**	**72 (8.4)**	**< 0.001**	**2.26 (1.50,3.41)**
Any major NDD	72 (29.5)	214 (25.1)	0.163	1.25 (0.91,1.72)
Risk level	*n* = 265	*n* = 906		
Risk to self	**217 (81.9)**	**169 (18.7)**	**< 0.001**	**19.72 (13.83,28.10)**
Risk to others	**132 (49.8)**	**261 (28.8)**	**< 0.001**	**2.45 (1.85,3.25)**

Notes: *n* values differ across variables due to missing data concerning gender (*n* = 43) and relevant factors; 5 young people identified as transgender and 1 young person as intersex and were not included in analyses; NDD = Neurodevelopmental Disorder (i.e., Learning disability, Autistic Spectrum Disorder, or Attention Deficit Hyperactivity Disorder (ADHD)); OR = odd ratios, CI = confidence intervals; Significant group differences and odds ratios are highlighted in bold

While the number of young men with any psychiatric diagnosis (372) was almost double the number of young women (214) with at least one, young women (214/244 or 87.7%) had a 9-fold increase in the odds of having a psychiatric diagnosis compared with young men (372/854 or 43.6%) and more than double the odds of having three or more psychiatric diagnoses (Table 1). Risk to self was identified in more than four out of five young women in secure care, a much higher rate than in young men (18.7%). Risk to others was also more commonly identified in young women, although this reflected the identification of low risk levels in YJS settings where the vast majority of young men were placed. In contrast, young women (89/182 or 48.9%) were less often considered a risk to others than young men in secure hospitals (69/90 or 76.7%, *p* < 0.001) while risk rates in secure welfare settings were comparable between females (26/52 or 50.0%) and males (15/28 or 53.6%, *p* = 0.761).

While the proportions and actual numbers of young men and young women with any or multiple psychiatric diagnoses is different, this, on its own, provides no information on the whether or not the pattern of specific mental health problems is the same by gender. This information is equally important as it relates to where an individual might best receive specific treatment and is key to effective service planning and provision.

**Fig. 2:**
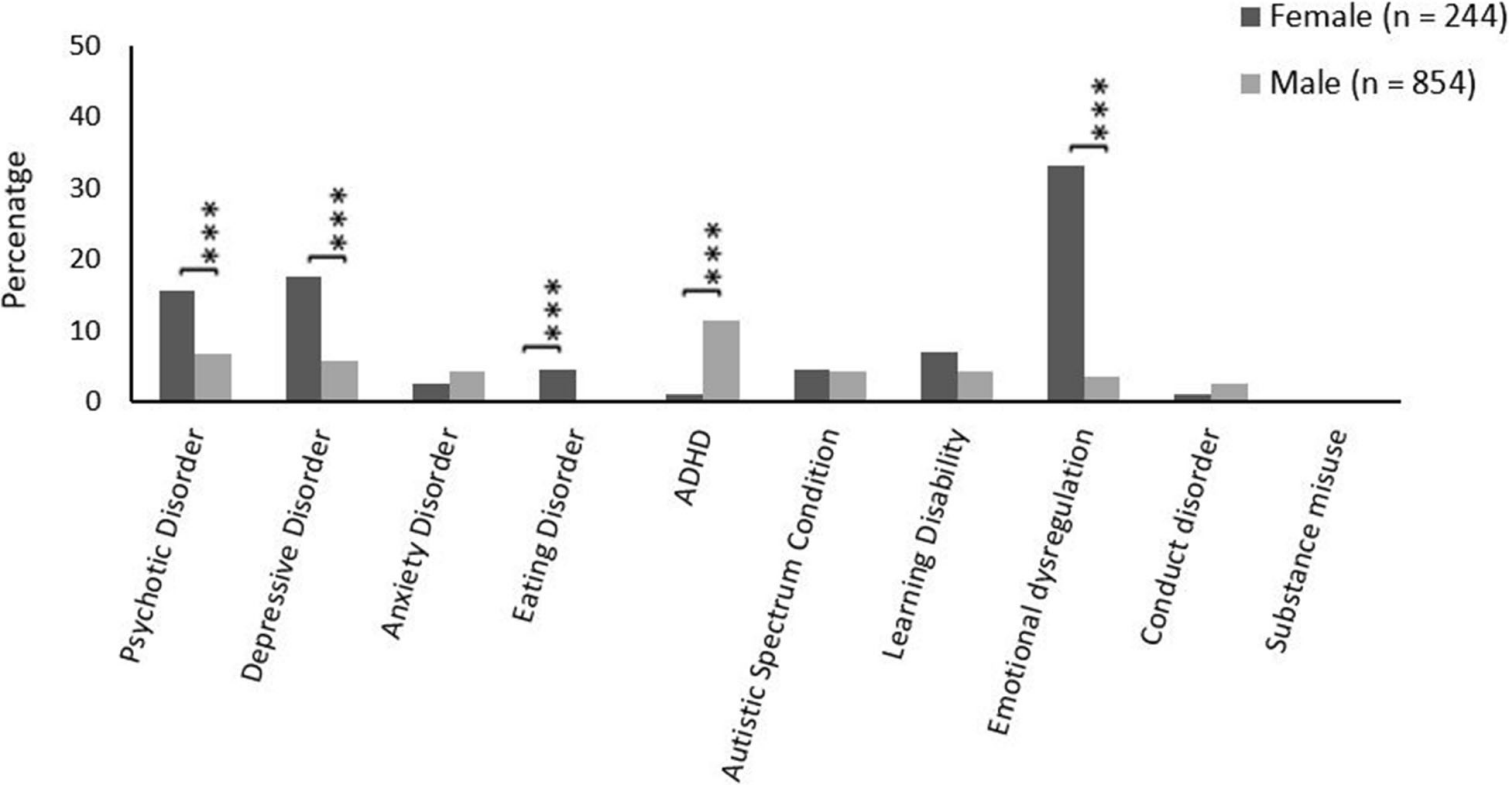
Primary (mental illness/neurodevelopmental disorder) diagnosis rates for young men and young women in secure care. Please note, 5 young people identified as transgender and 1 young person as intersex and were not included in analyses; gender and/or diagnosis data was missing for 218 young people in secure care; ADHD = Attention Deficit Hyperactivity Disorder; * Indicates significant group differences; ***p < 0.001

As Fig. 2 indicates, the populations of young men and young women differed significantly in terms of the likelihood of having a primary diagnosis of psychotic disorders, depressive disorders, eating disorders and emotional dysregulation. Young women had greater odds than young men of having psychotic disorders (15.6% vs 6.8%, OR = 2.53, 95% CI = 1.64, 3.92), depressive disorders (17.6% vs 5.9%, OR = 3.44, 95% CI = 2.22, 5.32), emotional dysregulation (33.2% vs 3.6%, OR = 13.19, 95% CI = 8.44, 20.63) and eating disorders (4.5% vs 0%, OR calculation N/A) but were less frequently diagnosed with ADHD (1.2% vs 11.4%, OR = 0.10, 95% CI = 0.03, 0.3).

### Patterns of placement by gender

**Fig. 3:**
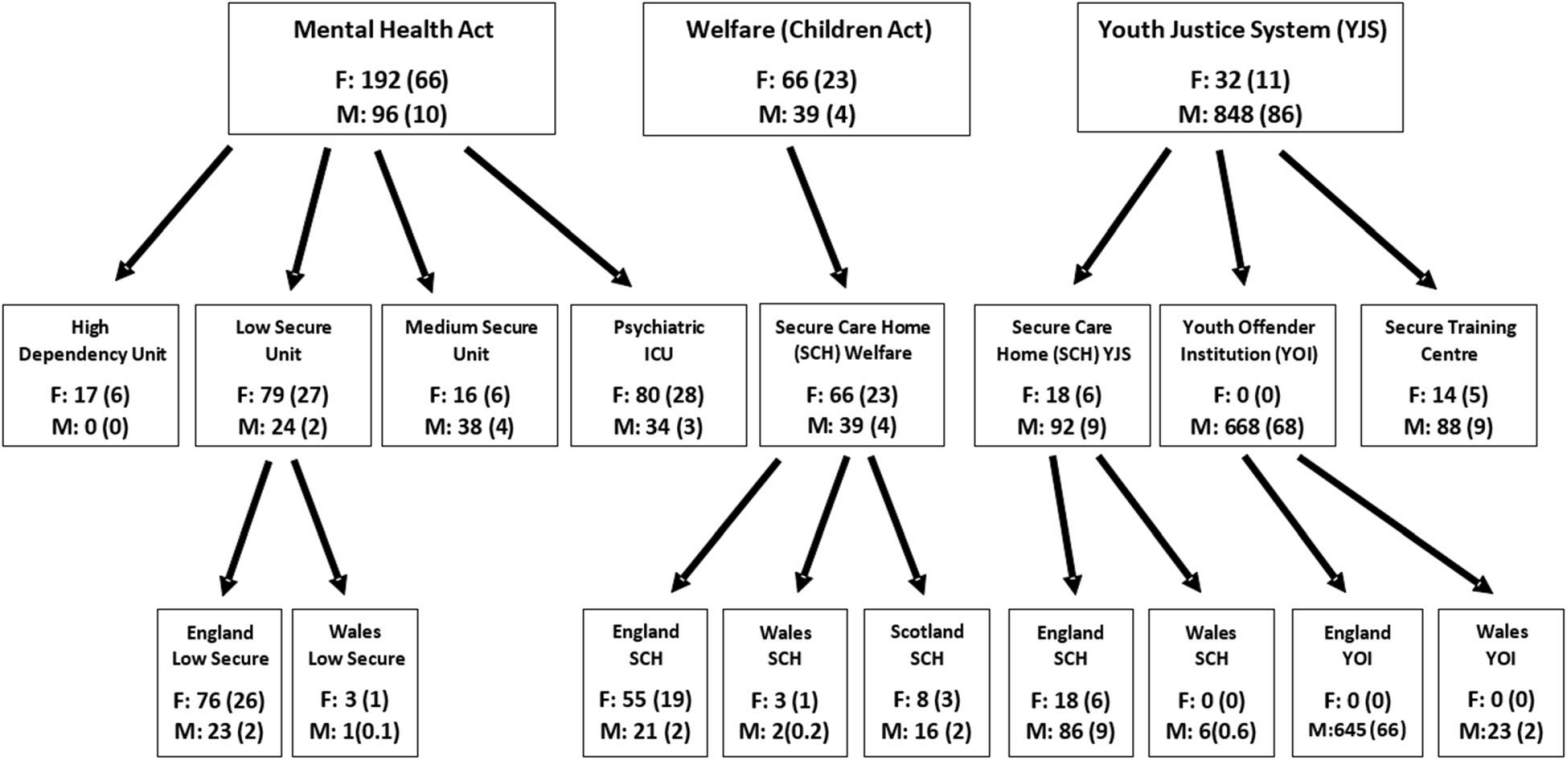
System Distribution: English young females and males (F:M) in secure care in Great Britain (*n* = 1273; female = 290, male = 983). Values represent frequencies (percentages). Please note, 5 young people identified as transgender (1 in England low secure, 2 in Psychiatric ICU, 1 in England SCH (Welfare), 1 in England SCH (YJS)), 1 young person as intersex (Psychiatric ICU); gender data was missing for 43 young people (not included in Figure)

As Fig. 3 below shows young men and young women are not equally represented in the 3 arms of the system of secure care. Most young women are in the mental health system (192, 66.2%) and a small number in welfare (66, 22.8%). In both these settings, there are more young women than men. The YJS contains the majority of all the young men (848, 86.3%), most of whom are in YOIs (668, 68.0%).

**Fig. 4:**
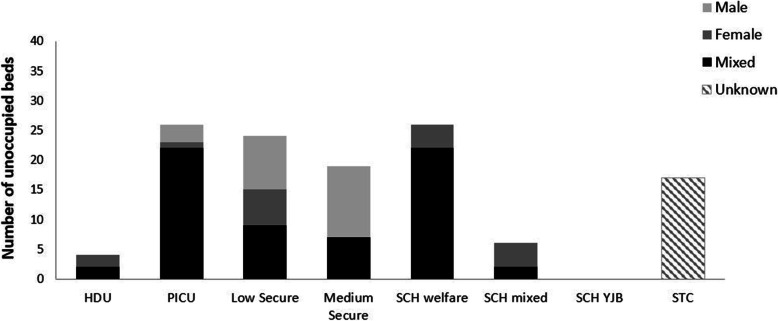
Number of unused secure placements (i.e., free beds) on 14 September 2016 according to type of unit. Please note, there were 17 unoccupied beds in STCs but the breakdown according to gender was not available; there were 254 unoccupied (male) beds in youth offender institutions (not shown on graph); HDU = High Dependency Unit, PICU = Psychiatric Intensive Care Unit, SCH = Secure Children’s Home, YJB = Youth Justice Board, STC = Secure Training Centre

This system is not full but the availability of placements varies by setting, level of security and gender (Fig. 4).

Given the variation in placement capacity across units by gender, and that the populations of young men and young women differ significantly in terms of their mental health profile, it is not particularly surprising to find different ratios of young men and young women across the system, as we demonstrated in Fig. 3. However, in and of themselves, these data do not tell us anything about the appropriateness of placements in terms of mental health needs. It warrants a further analysis to consider whether a young person of a particular gender, with a particular diagnosis is more or less likely to be in a particular kind of placement. The results of this analysis are in Table 2 below.

**Table 2:** Number (percentage) of female and male young people with different psychiatric diagnoses placed in Mental Health, Welfare and Youth Justice System legislative frameworks

	Psychiatric Diagnosis											
	Any diagnosis		Three or more diagnoses		Any major NDD							
	Females (*n* = 214)	Males (*n* = 372)	Females (*n* = 42)	Males (*n* = 72)	Females (*n* = 72)	Males (*n* = 214)						
	*n*(%)	*n*(%)	*n*(%)	*n*(%)	*n*(%)	*n*(%)						
Mental health	**184 (86.0)**	89 (23.9)***	**39 (92.9)**	37 (51.4)***	**62 (86.1)**	57 (26.6)***						
Welfare	**18 (8.4)**	12 (3.2)**	0 (0.0)	3 (4.2)	6 (8.3)	10 (4.7)						
Youth Justice System	**12 (5.6)**	271 (72.8)***	**3 (7.1)**	32 (44.4)***	**4 (5.6)**	147 (68.7)***						
	Primary Diagnosis											
	Psychosis		Emotional Dysregulation		Depression		Learning Disability		ASC		ADHD	
	Females (*n* = 38)	Males (*n* = 58)	Females (*n* = 81)	Males (*n* = 31)	Females (*n* = 43)	Males (*n* = 50)	Females (*n* = 17)	Males (*n* = 38)	Females (*n* = 11)	Males (*n* = 36)	Females (*n* = 3)	Males (*n* = 97)
	*n*(%)	*n*(%)	*n*(%)	*n*(%)	*n*(%)	*n*(%)	*n*(%)	*n*(%)	*n*(%)	*n*(%)	*n*(%)	*n*(%)
Mental health	**37 (97.4)**	42 (72.4)**	**70 (86.4)**	7 (22.6)***	**38 (88.4)**	9 (18.0)***	13 (76.5)	19 (50.0)	**10 (90.9)**	7 (19.4)***	1 (33.3)	2 (2.1)
Welfare	0 (0.0)	0 (0.0)	8 (9.9)	0 (0.0)	1 (2.3)	1 (2.0)	**3 (17.6)**	0 (0.0)*	1 (9.1)	1 (2.8)	**2 (66.7)**	8 (8.2)*
Youth Justice System	**1 (2.6)**	16 (27.6)**	**3 (3.7)**	24 (77.4)***	**4 (9.3)**	40 (80.0)***	**1 (5.9)**	19 (50.0)**	**0 (0.0)**	28 (77.8)***	**0 (0.0)**	87 (89.7)**

Notes: 5 young people identified as transgender and 1 young person as intersex and were not included in analyses; gender and/or diagnosis data was missing for 218 young people; F = female, M = male, NDD = Neurodevelopmental Disorder; ASD = Autistic Spectrum Condition, ADHD = Attention Deficit Hyperactivity Disorder; Significant gender differences (diagnosis rates in each legislative framework) after correction for multiple comparisons are highlighted in bold; *p < 0.05, **p < 0.01, ***p < 0.001

It is clear from this table that young men and young women with the same primary mental health diagnosis are frequently detained under different legislative frameworks. The welfare system detains very few young people with mental health problems. Young women with a mental health problem are more likely to be in hospital than young men (OR = 19.50, CI = 12.39, 30.69); this remains the case even where the young person has multiple (three or more) diagnoses (OR = 12.30, CI = 3.48, 43.44), which could be seen a marker of complexity. More than 70% of young men with a mental health problem are placed in the YJS, predominantly in YOIs, while almost half of the young men with more than three psychiatric diagnoses are placed in the YJS.

Data were then interrogated for diagnosis where the likely management, regardless of offending profile, would be in hospital (psychosis and Learning Disability) or not in hospital (ASC and ADHD). Significant gender differences in placement persisted (Table 2). A quarter of young men with primary psychosis (16/58 or 27.6%) but only one young woman (1/37 or 2.6%) were in the YJS: all the other young people with psychosis were in hospital. Half the young men with Learning Disability (19/38 or 50.0%) but only one young woman (1/17 or 5.9%) were in the YJS; most of the other young people with Learning Disability were in hospital (females 13/17 or 76.5%; males 19/38 or 50.0%). In addition, three quarters of young men with ASC (28/36 or 77.8%) were in the YJS and no young women (0/11) were. Young women with ASC (10/11 or 90.9%) were almost all in hospital. Nine out of ten young men with ADHD (87/97 or 89.7%) were in the YJS. Close to 90% of young women with primary emotional dysregulation (70/81 or 86.4%) or depression (38/43 or 88.4%) were placed in hospital. In contrast, only about a fifth of young men with the same problems (16/81 or 19.8%) were in hospital, with most placed in the YJS.

While across all secure care institutions, a mental illness diagnosis was significantly less frequent in BAME young men (114/331 or 34.4%) than in white young men (217/434 or 50.0%; χ^2^ = 18.52; *p* < 0.001; OR = 0.53, 95% CI = 0.39,0.71), the proportions of BAME and white young men with a primary mental illness diagnosis (excluding ADHD) placed in the YJS were comparable (BAME 57/81 or 70.4% vs. white 106/167 or 63.5%; χ^2^ = 1.15; *p* = 0.283). There was no difference in rates of any mental illness diagnosis between BAME (27/31 or 87.1%) and white young women (154/178 or 86.5%, *p* = 0.999) nor any difference in the proportions of BAME and white young females with a diagnosis (excluding ADHD) placed in secure hospital (BAME 21/26 or 80.8% vs. white 133/152 or 87.5%; χ^2^ = 0.86; *p* = 0.353).

## Discussion

This study explored a simple question i.e. whether the system of detention for young people responded in the same way to mental health problems in its population, regardless of the gender of the young person concerned. Our results show not only that the frequency and type of mental disorder vary by gender in the sample population as a whole but that their distribution varies by gender across the types of secure provision. We have found significant differences in the pattern of placement of young men and women, when their serious mental health needs are the same. This raises serious questions about the equity of the process of assessment and placement for young men and young women. Overall, the frequency of mental disorders found in young men and young women in welfare settings is low so this discussion focuses mainly on the mental health and youth justice components of the system of detention.

Patterns of morbidity have previously been found to differ by gender within individual welfare [10], secure hospital [14] and youth justice [15, 22] settings but an uncritical approach has been taken to findings of different but significant morbidity in young men and young women in that there are frequent calls for improvement in service provision but scant attention to processes of entry and exit to secure care. We have found no obviously comparable data to ours, which for the first time highlights inequitable processing of young men and young women, either in the UK or in other jurisdictions. This paper adds to existing knowledge by emphasizing the importance of real world, jurisdiction specific process issues in understanding why gender distinctions may emerge.

### Explanations of placement patterns

The fact that young men and women with the same mental health problems do not have the same placement patterns matter because different settings provide different levels of care by virtue of regime ethos and availability of psychiatric services [23].

Several explanations need to be considered. First, the offending history of the young person may constrain or determine placement possibilities. Although young men were more likely to have convictions resulting in imprisonment, even grave offending is no bar to accessing the mental health components of the detention system, pre- or post-conviction. Medium secure hospital units are designed to care for young people who may have committed such offences and who have identifiable mental health needs suitable for hospital-based assessment and treatment.

Second, the picture of placements obtained may reflect the availability of suitable placements in different parts of the system for young men and young women. Parts of the youth justice system had significant spare capacity, most obviously for young men at the time of data collection, other parts had no capacity; secure mental health provision had capacity, which varied by the level of security. There were no female medium secure beds available but there were male medium secure beds and beds to which young men could be admitted at all other levels of security. The widespread availability of beds suitable for either young men or young women (designated as mixed in Fig. 3) suggests flexibility in the system, although this analysis takes no account of geography or of individuals’ specific needs which might make placement with young people of the opposite sex unsuitable e.g. trauma issues, sexual offending.

Third, psychiatric disorders may look so different in young men and young women that their presentation, under the same diagnostic label, warrants a different response. This is contrary to the nature of diagnosis which should determine subsequent clinical management independent of gender. Having said that, the nature of comorbidity and levels of violence and aggression linked to particular mental disorders may alter the care plan.

Fourth, the place of detention may be determined by risk to self rather than risk to others and that might affect young women disproportionately, as it does adult women [25] where secure hospitalisation is often used solely for risk to self. Rates of self-harm among young offenders [26] are at a 5 year high, with rates among young women in the YJS ten times that of young men; this may invite a different institutional response, including possible transfer to hospital.

Fifth, some of the diagnoses are only determined post placement; had they had been known earlier they may have led to a different decision. Diagnoses may only come into play late in the day, transfers to hospital take time and information is not presented to court that might allow for a non-custodial sentence.

Sixth, decision making within a complex system of placement may be unconsciously or consciously affected by the gender of the young person. How that could happen in these domains is poorly understood despite increasing recognition of the role of unconscious biases of different kinds within both the mental health and criminal justice system for adults and ordinary places of work; the introduction of mandatory diversity training within the public sector in the UK does not guarantee the eradication of such bias. Sharpe and Gelsthorpe [27] describe the complexity and changing nature of youth justice processes which aim to be gender neutral or gender differentiating, highlighting the difficulty of avoiding gender stereotyping but responding to gender specific needs. Similarly complex processes related to ethnicity are recognised in the UK and elsewhere [4, 28] as contributing to the over representation of young black men in the youth justice system. The intersectionality of gender and ethnicity creates further difficulties of interpretation in our data. It may be important to resist easy conclusions, which may well be stretching the reach of our data, about differential rates of mental illness in young men of different ethnicities across the system as a whole, particularly when this is not seen in young women.

### Consequences of placement patterns

Explanations of the current pattern of placement of young men and young women with mental health problems is necessarily tentative, most obviously where process issues may be thought relevant. But it is still reasonable to consider the consequences, for individuals with specific mental health problems, that follow from their placement type.

There is no doubt that the youth justice system has seen rising levels of violence, use of restraint and self-harm [26, 29] with some units investigated for staff brutality [30]. Even without this, the nature of regimes in the youth justice system arguably result in them being unsuitable places for young men with psychotic illnesses, those who are vulnerable by virtue of ASC or LD and where those with untreated ADHD might find it difficult to cope. In addition, they may find that they cannot engage in rehabilitative programmes designed for those without cognitive (levels of intelligence, poor concentration) or emotional problems (abnormal perceptions, lacking interpersonal skills) and where no reasonable adjustments have been made.

The issues generated by the nature of the regimes is compounded by the relative absence of clinical staff. Even when teams are fully staffed, they may lack specialist expertise and are few in number [23]. The YJS lacks treatment options (e.g. Dialectical Behaviour Therapy, Sensory interventions for autism) found elsewhere and, in this instance, young men are clearly disadvantaged by comparison with young women.

However, looked at another way, the young women are vulnerable to the stigma of long term hospitalisation. For young women who find themselves on hospital orders early in adolescence that have no end date, institutionalisation may operate against any treatment gains or normal maturation. Where the primary psychiatric problem e.g. ASC, does not readily lend itself to hospital based treatment individuals may find it hard to progress; welfare options might be more appropriate and are clearly not currently being used. Key to the issue of fairness is that young women’s criminal careers are often brief, more frequently than young men’s and long periods of detention, however well intentioned, may look unfair [31]. The advent of Secure Stairs (with its emphasis on promoting psychological models of distress/anger to staff and young people and addressing these in a well-resourced, multi-agency way) [32] in the YJS might be more useful with regard to emerging personality disorders and emotional dysregulation than hospitalisation. However, the downsizing of penal custody for young women, very much in line with the Bangkok rules [33] means this is unlikely to be a realistic or desirable way forward. Again, the development of improved community and/or secure welfare options for this group, who are often emerging from the care system anyway [6] could be a better option.

### Difference or discrimination?

The Lammy Review [4] (p11) suggested that “scrutiny is the best route to fair treatment”, a remark that could as well apply to gender as to ethnicity. While this study has identified areas of concern within the system of detention for young people, issues that pull young men and young women in different directions, it sheds limited light on how and why this happens. However, it cannot be right that your gender determines the likelihood of you being in the best place to get the right treatment for your mental health problems as this study suggests. The imperative of fair treatment demands that the data gap in decision making process outlined above is addressed. This means understanding how decision making happens en route to placement and who decides on placement. Lammy also paid attention to the interplay between young men and the youth justice system where their decisions also influenced their own outcomes; equally, it would be important to understand if and how the stigma of mental health problems may make young people reluctant to seek appropriate help.

## Conclusions

There is a need not only for the current ongoing monitoring of a system that has such enormous implications for a young person’s life chances but also regular, rigorous interrogation of routine data. Only with transparency built into both data collection on individuals’ characteristics and on the enactment of process will it be possible to protect troubled young men and women from unlawful, gender based disadvantage caused by discrimination, individual or institutional, inadvertent or deliberate.

### Limitations

This study is cross sectional and the complex system from which it is drawn is subject to change.

Diagnostic information was obtained from professionals of different backgrounds with different conceptual frameworks and at times derived by the study team from needs based information provided.

Limited information on risk to self and others was available.

Other factors than treatment availability are relevant to the suitability of placement e.g. distance from home.

The study design was not intended to capture decision making processes which may underlie some of the gender differences in placement pattern identified.

## Data Availability

The datasets used and/or analysed during the current study are available from the corresponding author on reasonable request.
